# Hearing Loss in Stranded Odontocete Dolphins and Whales

**DOI:** 10.1371/journal.pone.0013824

**Published:** 2010-11-03

**Authors:** David Mann, Mandy Hill-Cook, Charles Manire, Danielle Greenhow, Eric Montie, Jessica Powell, Randall Wells, Gordon Bauer, Petra Cunningham-Smith, Robert Lingenfelser, Robert DiGiovanni, Abigale Stone, Micah Brodsky, Robert Stevens, George Kieffer, Paul Hoetjes

**Affiliations:** 1 College of Marine Science, University of South Florida, St. Petersburg, Florida, United States of America; 2 Mote Marine Laboratory, Sarasota, Florida, United States of America; 3 Department of Biology, Portland State University, Portland, Oregon, United States of America; 4 Chicago Zoological Society, c/o Mote Marine Laboratory, Sarasota, Florida, United States of America; 5 Division of Social Sciences, New College of Florida, Sarasota, Florida, United States of America; 6 Marine Mammal Conservancy, Key Largo, Florida, United States of America; 7 Riverhead Foundation for Marine Research and Preservation, Riverhead, New York, United States of America; 8 Clearwater Marine Aquarium, Clearwater, Florida, United States of America; 9 Dolphins Plus, Key Largo, Florida, United States of America; 10 Southern Caribbean Cetacean Network (SCCN), Bapor Kibra, Curaçao, Netherlands Antilles; 11 Department of Environment and Nature, Willemstad, Curaçao, Netherlands Antilles; University of Lethbridge, Canada

## Abstract

The causes of dolphin and whale stranding can often be difficult to determine. Because toothed whales rely on echolocation for orientation and feeding, hearing deficits could lead to stranding. We report on the results of auditory evoked potential measurements from eight species of odontocete cetaceans that were found stranded or severely entangled in fishing gear during the period 2004 through 2009. Approximately 57% of the bottlenose dolphins and 36% of the rough-toothed dolphins had significant hearing deficits with a reduction in sensitivity equivalent to severe (70–90 dB) or profound (>90 dB) hearing loss in humans. The only stranded short-finned pilot whale examined had profound hearing loss. No impairments were detected in seven Risso's dolphins from three different stranding events, two pygmy killer whales, one Atlantic spotted dolphin, one spinner dolphin, or a juvenile Gervais' beaked whale. Hearing impairment could play a significant role in some cetacean stranding events, and the hearing of all cetaceans in rehabilitation should be tested.

## Introduction

Odontocete cetaceans (the toothed whales) use high frequency echolocation sounds for navigation and foraging and are well-known for possessing high frequency hearing abilities [1]–[2]. Many species strand in coastal areas, as individuals or sometimes in large mass stranding events [3]. The causes of dolphin and whale stranding are often difficult to determine. Here we show profound high-frequency hearing loss in three species of cetaceans that had stranded, which suggests that hearing impairment could be a causative factor for some stranding events.

Hearing measurements in captive cetaceans are often performed with behavioral conditioning, where the dolphin is trained to press a response paddle or vocalize when it hears a test signal [1], [4]–[6]. Behavioral hearing tests cannot be easily performed on stranded cetaceans. Stranded dolphins often reside in rehabilitation facilities for short periods of time, and training of stranded dolphins that are likely to be returned to the wild is discouraged by federal wildlife management agencies. Auditory evoked potential (AEP) methods, which are commonly used to measure hearing in human infants [7], have been used to measure hearing in captive and wild cetaceans [8]–[10]. AEPs provide a rapid means to assess hearing and yield comparable results to behavioral measures [11]–[14]. A further advantage is that AEPs provide the means to screen free-ranging populations of cetaceans if individuals are able to be handled briefly.

## Results

Severe (∼70–90 dB loss) to profound (>90 dB loss) hearing loss was found in four out of seven (57%) of the stranded bottlenose dolphins (*Tursiops truncatus*) and 5 out of 14 (36%) rough-toothed dolphins (*Steno bredanensis*) examined in this study (definitions based on [15]) (Table 1; detailed information on each animal can be found in Table S1). This finding is in stark contrast to results of population-level hearing evaluations of bottlenose dolphin in Sarasota Bay, Florida, during capture-release health assessments [16] that have shown a general absence of hearing loss across more than 60 dolphins of a variety of ages and both sexes [17]. Profound hearing loss was also found in one stranded short-finned pilot whale (*Globicephala macrorhynchus)*. No hearing loss was found in stranded Rissos's dolphins (*Grampus griseus*) from three different stranding events, two pygmy killer whales (*Feresa attenuata*) from a single stranding event, an Atlantic spotted dolphin (*Stenella frontalis*), and a spinner dolphin (*Stenella longirostris*; Table 1). The hearing of the Risso's dolphins were similar to the hearing measured from a stranded Risso's dolphin previously reported [18]. The audiograms of species with normal hearing will be reported elsewhere.

**Table 1:** Summary of odontocete audiogram results.

Species	Common Name	Number Stranded Animals Tested	Number with Hearing Loss
*Steno bredanensis*	Rough-toothed dolphin	14	5
*Tursiops truncatus*	Bottlenose dolphin	7*	4*
*Grampus griseus*	Risso's dolphin	7	0
*Feresa attenuata*	Pygmy killer whale	2	0
*Stenella longirostris*	Spinner dolphin	1	0
*Stenella frontalis*	Atlantic spotted dolphin	1	0
*Globicephala macrorhynchus*	Short-finned pilot whale	1	1
*Mesoplodon europaeus* [*13*]	Beaked whale	1	0

*One bottlenose dolphin (Filly) became entangled in fishing gear and was rescued. This dolphin had high frequency hearing loss.

**Figure 1:**
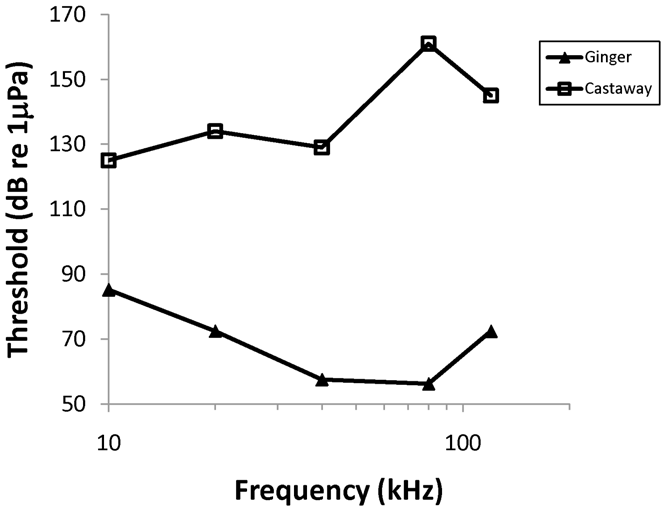
Audiogram of MML0807 (Ginger; filled triangles), a juvenile bottlenose dolphin with normal hearing, and MML FB303 (Castaway; open squares), an adult bottlenose dolphin with hearing loss. For Castaway, no response was detected at any frequency indicating that there was at least 40–100 dB hearing loss across all frequencies tested, and no response could be detected in response to click trains at 164 dB_peak_ re 1 µPa.

**Figure 2:**
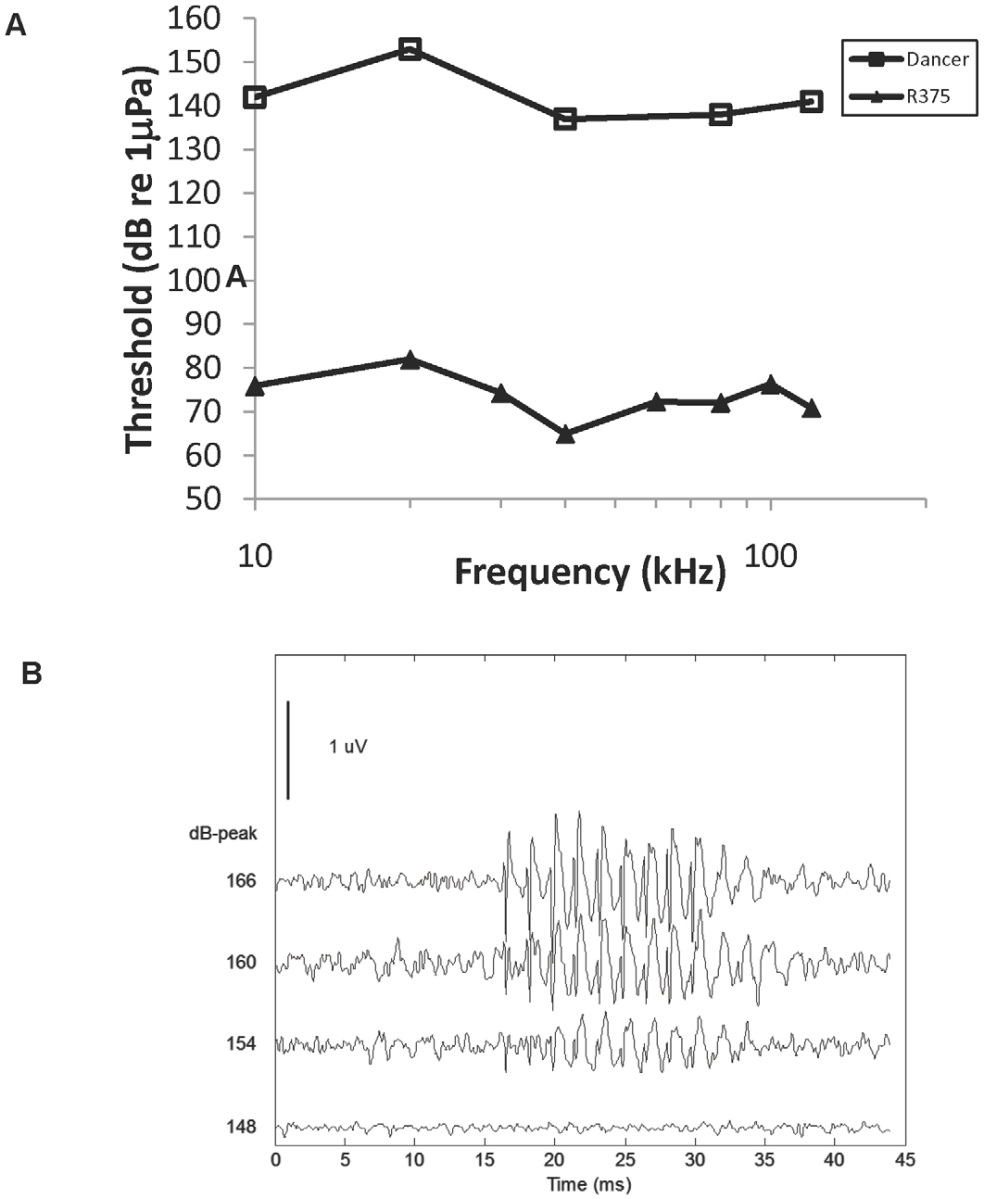
Results of hearing tests on two stranded rough-toothed dolphins. A. Audiogram of R375 (filled triangles), a normal hearing rough-toothed dolphin, and highest level tested for Dancer (open squares) rough-toothed (open squares) for which no response was detected at any frequency). For Dancer, there was at least a 70 dB hearing loss across all frequencies tested. B. Click-train (600 Hz click rate) evoked potential traces from the hearing-impaired rough-toothed dolphin (Dancer) at four stimulus levels. The click train sound starts at 15 ms. The lowest level detected was about 154 dB_peak_ re 1 µPa.

The audiograms of two stranded bottlenose dolphins are shown in Figure 1 to illustrate the degree of hearing loss that has been measured in stranded dolphins; in the case of the dolphin named “Castaway” there was at least 105 dB loss at 80 kHz and no auditory evoked potentials were detected at any frequency tested from 10 kHz to 120 kHz. In the rough-toothed dolphin named “Dancer”, no response was seen to the loudest tone pips that could be generated (137–153 dB re 1 µPa SPL), but an evoked potential could be seen in response to a click at high sound levels (154 dB re 1 µPa_peak_) (Figure 2). This audiogram shows at least 70 dB hearing loss in a rough-toothed dolphin (Figure 2). There was no statistical relationship between dolphin age group and hearing loss (Fisher Exact Test p = 1.000), when including all dolphins and grouping calves and subadults, because of the low sample size of calves (Table 2). However, none of the calves showed hearing loss, but 42% of the subadults had hearing loss. Five of the dolphins with profound hearing loss were males and five were females.

**Table 2:** Summary of hearing loss for all dolphins by age category.

	Normal	Hearing Loss	Total
Adult	13	5	18
Subadult	7	5	12
Calf	5	0	5

## Discussion

There are five main contributing factors to hearing loss in mammals: intense chronic noise, transient intense noise (e.g. explosions), age-related hearing loss (presbycusis), congenital hearing impairment, and ototoxic drug treatment [19]. We do not know the noise exposure history of any of these dolphins. Based on the locations of stranding, it is possible that some of them have been exposed to chronic noise from boating and shipping, while for others this is unlikely. Experiments with odontocetes have shown that high levels of exposure are needed to induce temporary threshold shifts [20]–[24]. One of the stranded bottlenose dolphins (Caesar II) with high frequency hearing loss was assumed to be relatively old based on his lack of teeth, which can wear down and/or fall out with age. Age-related hearing loss has been reported in some captive dolphins [25]-[28], but not commonly in wild dolphins [17]. It is possible that wild dolphins with hearing loss have lower survival than dolphins with normal hearing.

It is likely that hearing loss was congenital in some of these stranding cases, especially two of the rough-toothed dolphins (Dancer and Vixen), which were estimated to each be approximately two years old at the time of stranding. Congenital hearing impairment in humans is not uncommon. In one study, 113 out of 52508 (about 2 out of 1,000) newborns screened in Texas had hearing loss [7]. In addition to genetic factors related to hearing loss, one extremely important, unexplored mechanism may be the impacts of chemical pollution on hearing. Cetaceans, particularly odontocetes, have been shown to accumulate very high levels of polychlorinated biphenyls (or PCBs), which can be maternally transferred to offspring [29]–[30]. Evidence from studies with rats suggests that these chemicals may affect how hearing develops. In fact, it has been shown that developmental exposure of rats to PCBs results in severe hearing loss [31]. It is not known how these or other contaminants might affect ear development in cetaceans.

**Figure 3:**
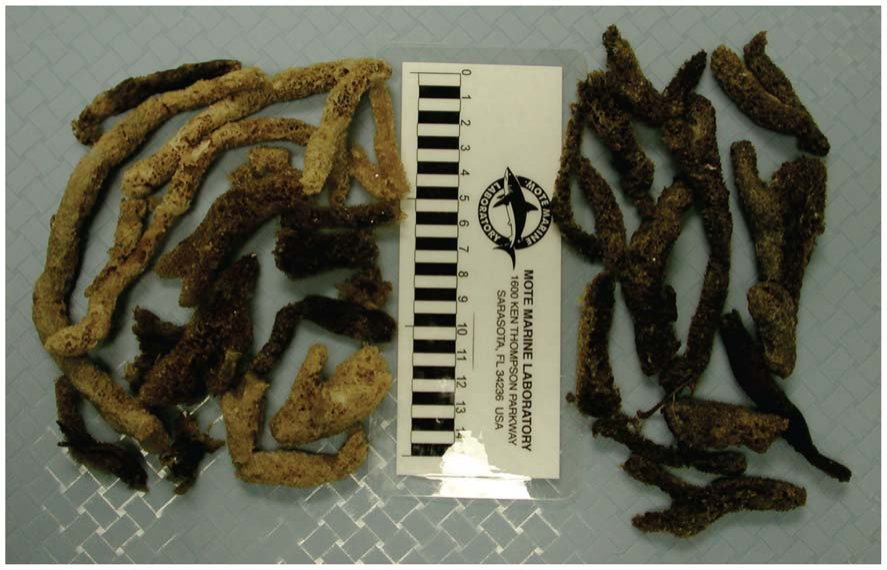
Image of sponges removed from the stomach of Vixen, a stranded rough-toothed dolphin with profound hearing loss.

Some odontocetes have social systems with strong mother-calf bonds where the mother can support the calf for years through lactation [32]. While dolphins rely heavily on echolocation for navigation and foraging, it is possible that a dependent calf with congenital hearing loss could survive with the help of its mother for a long period. Once it is weaned, however, it would not be able to forage successfully on its own. Indeed, both of the young rough-toothed dolphins that stranded were found to have consumed sponges, possibly indicating an inability to catch squid and fishes (Figure 3). A previous case of a deaf bottlenose dolphin, which was captured in the wild, was reported healthy at the time of capture, which suggests that it is possible for a deaf dolphin to survive in a social group [25].

Many of the stranded dolphins in this study were being treated for various infections with antibiotics and antifungals, including the oral administration of gentamicin sulfate and itraconazole and intramuscular injections of amikacin sulfate. Aminoglycoside antibiotics, including gentamicin and amikacin, are known to cause cochlear hair cell death in mammals [33]. This mechanism has been suggested as a potential cause of hearing loss in captive marine mammals [27]. While drug administration cannot be ruled out as a cause of hearing loss, it is unlikely as some of the hearing tests were conducted soon after stranding. In addition, dolphins that showed hearing loss were treated with similar drug dosages as other animals that did not show hearing deficits. Hearing tests were conducted both before and after drug treatment with five Risso's dolphins, none of which showed an effect on hearing. In future stranding events, it is crucial that hearing measurements be performed both before and after any drug treatment. Rehabilitation will not be successful if drug treatments result in significant high frequency hearing loss. One recent report of aspergillosis in a stranded harbor porpoise showed infection of the otic region [34]. The high incidence of hearing impairment in stranded odontocetes also prompts us to argue that hearing screening should be part of the standard veterinary examination of stranded cetaceans.

## Methods

All research was approved by the IACUC of the University of South Florida and Mote Marine Laboratory with permit authorization from NOAA. Hearing tests were conducted under NOAA permits #932-1489-06 (letter Sep 1, 2004) for *Steno bredanensis* at MML; #932-1489-06 (letter Apr 08, 2005) for *Grampus griseus* Riverhead Foundation; #932-1489-06 (letter April 20, 2005) for *S. bredanensis* at Marine Mammal Conservancy; #932-1489-08 (letter Aug 24, 2005) for *S. bredanensis* at Gulf World; #932-1489-08 (letter Aug 14, 2005) for *Grampus griseus* at MML; #1053-1825-00 (Nov 21, 2006) for all other dolphins. Hearing tests were conducted either in the water or on land, depending on the situation at the stranding facility (e.g., whether handlers could stand in the holding tanks or pens, or the number of other animals in the holding tanks). In all cases, a jawphone consisting of an ITC-1042 piezoceramic transducer embedded in a RTV silicone suction cup was placed on the lower jaw fat pad to deliver sound stimuli. Sound levels were calibrated underwater by measuring the sound 10 cm from the jawphone in a free field using a Reson TC4032 hydrophone. These underwater calibrations were used as the calibrations for the in-air tests. Background noise levels in test tanks were typically <45 dB re 1 µPa^2^/Hz at frequencies above 20 kHz. Noise levels in field test locations, which were shallow beaches (Florida Bay at the Marine Mammal Conservancy and the Caribbean Sea at Curacao Seaquarium), were about 85 dB re 1 µPa^2^/Hz at frequencies above 20 kHz.

Evoked potentials were measured in response to amplitude modulated (AM) tone pips. The AM rate was either 600 Hz or 1000 Hz, as these rates were found to yield large evoked potentials. Click evoked potentials were also measured for some dolphins. Clicks were generated by sending a 0.1 ms square wave into the transducer, which generated a click with a peak frequency of approximately 80–120 kHz. Evoked potentials were measured in response to click trains delivered at 600 Hz and 1000 Hz. All signals were generated by a Tucker-Davis Technologies (TDT) RX6 real-time processor with a 260 kHz sample rate.

Silver chloride electrodes embedded in suction cups were used to measure evoked potentials after 10,000× amplification through a differential amplifier (TDT HS4-DB4). The recording electrode was placed about 5–10 cm behind the blowhole, and a reference electrode was placed approximately 40 cm behind the blowhole. The ground electrode was placed in the water for in-water tests, or on the back of the dolphin for in-air tests.

Evoked potentials were averaged in response to up to 1000 presentations of the acoustic stimulus (TDT BioSig software). Fewer presentations were used when a response at one level was apparent. AEP signals were analyzed in a custom MATLAB program (BSlab) which performed a 2441-point FFT (resulting in a 10 Hz resolution) and measured the signal-to-noise ratio at the modulation frequency of the AM signals. A signal was considered to be present if the AEP signal was at least 3 dB above the background noise (either measured from 12 adjacent frequency bins, or from a portion of the signal in which sound was not presented). This is equivalent to the F-test with an alpha of at least 0.01 [35]. The threshold was defined as the lowest signal level that yielded a detectable AEP at the sound modulation frequency.

## Supporting Information

Table S1 Summary of odontocete audiogram results. One bottlenose dolphin, MML0701 (Filly), became entangled in fishing gear and was rescued. MML = Mote Marine Laboratory; MMC = Marine Mammal Conservancy; CMA = Clearwater Marine Aquarium; Riverhead = Riverhead Foundation; Curacao = Curacao Seaquarium. Dolphins with severe to profound hearing loss are also indicated in bold type.
(0.02 MB DOCX)

## References

[1] Johnson CS, Tavolga W (1967). Sound detection thresholds in marine mammals.. Marine bio-acoustics.

[2] Au W (1993). The Sonar of Dolphins..

[3] Odell D (1987). The mystery of marine mammal strandings.. Cetus.

[4] Hall JD, Johnson CS (1972). Auditory thresholds of a killer whale *Orcinus orca*. Linneaus.. J Acoust Soc Am.

[5] FT Awbrey FT, JA Thomas JA, Kastelein RA (1988). Low frequency underwater hearing sensitivity in belugas (*Dephinapterus leucas*).. J Acoust Soc Am.

[6] Kastelein RA, Hagedoorn M, Au WWL, de Haan D (2003). Audiogram of a striped dolphin (*Stenella coeruleoalba*).. J Acoust Soc Am.

[7] Finitzo T, Albright K, O'Neal J (1998). The newborn with hearing loss: detection in the nursery.. Pediatrics.

[8] Popov VV, Supin AY (1990). Auditory brainstem responses in characterization of dolphin hearing.. J Comp Physiol A.

[9] Supin AY, Popov VV (1995). Envelope-following response and modulation transfer function in the dolphin's auditory system.. Hear Res.

[10] Dolphin WF, Au WW, Nachtigall PE, Pawloski J (1995). Modulation rate transfer functions to low-frequency carriers in three species of cetaceans.. J Comp Physiol A.

[11] Szymanski MD, Bain DE, Kiehl K, Pennington S, Wong S (1999). Killer whale (*Orcinus orca*) hearing: Auditory brainstem response and behavioral audiograms.. J Acoust Soc Am.

[12] Yuen MML, Nachtigall PE, Breese M, Supin AY (2005). Behavioral and auditory evoked potential audiograms of a false killer whale (*Pseudorca crassidens*).. J Acoust Soc Am.

[13] Cook MLH, Varela RA, Goldstein JD, McCulloch SD, Bossart GD (2006). Beaked whale auditory evoked potential hearing measurements.. J Comp Phys A.

[14] Houser DS, Finneran JJ (2006). A comparison of underwater hearing sensitivity in bottlenose dolphins (*Tursiops truncatus*) determined by electrophysiological and behavioral methods.. J Acoust Soc Am.

[15] Clark JG (1981). Uses and abuses of hearing loss classification.. ASHA.

[16] Wells RS, Rhinehart HL, Hansen LJ, Sweeney JC, Townsend FI (2004). Bottlenose dolphins as marine ecosystem sentinels: Developing a health monitoring system.. EcoHealth.

[17] Cook MLH (2006). Behavioral and Auditory Evoked Potential (AEP) Hearing Measurements in Odontocete Cetaceans..

[18] Nachtigall PE, Yuen MML, Mooney TA, Taylor KA (2005). Hearing measurements from a stranded infant Risso's dolphin, *Grampus griseus*.. J Exp Biol.

[19] Tarter SK, Robins TG (1990). Chronic noise exposure, high-frequency hearing loss, and hypertension among automotive assembly works.. J Occupat Med.

[20] Finneran JJ, Schlundt CE, Dear R (2002). Temporary shift in masked hearing thresholds in odontocetes after exposure to single underwater impulses from a seismic watergun.. J Acoust Soc Am.

[21] Nachtigall PE, Pawloski JL, Au WWL (2003). Temporary threshold shifts and recovery following noise exposure in the Atlantic bottlenose dolphin *Tursiops truncatus*.. J Acoust Soc Am.

[22] Nachtigall PE, Supin AY, Pawloski JL, Au WWL (2004). Temporary threshold shifts after noise exposure in the bottlenose dolphin *Tursiops truncatus* measured using auditory evoked potentials.. Mar Mamm Sci.

[23] Finneran JJ, Carder DA, Schlundt CE, Ridgway SH (2005). Temporary threshold shift in bottlenose dolphins (*Tursiops truncatus*) exposed to mid-frequency tones.. J Acoust Soc Am.

[24] Lucke K, Siebert U, Lepper PA, Blanchet MA (2009). Temporary shift in masked hearing thresholds in a harbor porpoise (*Phocoena phocoena*) after exposure to seismic airgun stimuli.. J Acoust Soc Am.

[25] Ridgway SH, Carder DA (1997). Hearing deficits measured in some *Tursiops truncatus*, and discovery of a deaf/mute dolphin.. J Acoust Soc Am.

[26] Brill RL, Moore PWB (2001). Assessment of dolphin (*Tursiops truncatus*) auditory sensitivity and hearing loss using jawphones.. J Acoust Soc Am.

[27] Houser DS, Finneran JJ (2006). Variation in the hearing sensitivity of a dolphin population obtained through the use of evoked potential audiometry.. J Acoust Soc Am.

[28] Houser DS, Gomez-Rubio A, Finneran JJ (2008). Evoked potential audiometry of 13 Pacific bottlenose dolphins (*Tursiops truncatus gilli*).. Mar Mamm Sci.

[29] Ross PS, Ellis GM, Ikonomou MG, Barrett-Lennard LG, Addison RF (2000). High PCB concentrations in free-ranging Pacific killer whales, Orcinus orca: effects of age, sex and dietary preference.. Mar Pollut Bull.

[30] Wells RS, Tornero V, Borrell A, Aguilar A, Rowles TK (2005). Integrating life-history and reproductive success data to examine potential relationships with organochlorine compounds for bottlenose dolphins (Tursiops truncatus) in Sarasota Bay, Florida.. Sci Total Environ.

[31] Goldey ES, Crofton KM (1998). Thyroxine replacement attenuates hypothyroxinemia, hearing loss, and motor deficits following developmental exposure to Aroclor 1254 in rats.. Toxic Sci.

[32] Brodie PF (1969). Duration of lactation in Cetacea: An indicator of required learning?. Amer Mid Nat.

[33] Matz GJ, Lerner SA (1980). Aminoglycoside ototoxicity.. Am J Otolaryng.

[34] Prahl S, Jepson PD, Sanchez-Hanke M, Deaville R, Sibert U (2010). Aspergillosis in the middle ear of a harbour porpoise (*Phocoena phocoena*): a case report.. Mycoses.

[35] Dobie RA, Wilson MJ (1996). A comparison of t test, F test, and coherence methods of detecting steady-state auditory evoked potentials, distortion-product otoacoustic emissions, or other sinusoids.. J Acoust Soc Am.

