# Supplementary material for: Hearing Loss in Stranded Odontocete Dolphins and Whales
Source: PLoS One. 2010 Nov 3;5(11):e13824. doi: 10.1371/journal.pone.0013824 (PMC2972210; doi:10.1371/journal.pone.0013824)
Supplement: Table S1 — Summary of odontocete audiogram results. One bottlenose dolphin, MML0701 (Filly), became entangled in fishing gear and was rescued. MML = Mote Marine Laboratory; MMC = Marine Mammal Conservancy; CMA = Clearwater Marine Aquarium; Riverhead = Riverhead Foundation; Curacao = Curacao Seaquarium. Dolphins with severe to profound hearing loss are also indicated in bold type. (0.02 MB DOCX) [file pone.0013824.s001.docx]

Table S1. Summary of odontocete audiogram results. One bottlenose dolphin, MML0701 (Filly), became entangled in fishing gear and was rescued. MML=Mote Marine Laboratory; MMC=Marine Mammal Conservancy; CMA=Clearwater Marine Aquarium; Riverhead=Riverhead Foundation; Curacao=Curacao Seaquarium. Dolphins with severe to profound hearing loss are also indicated in bold type.

| **Species** | **Name** | **Location** | **Environment** | **Date Stranded** | **Date Tested** | **Sex** | **Age Group** | **Severe or Profound Hearing Loss** |
| --- | --- | --- | --- | --- | --- | --- | --- | --- |
| *Feresa attenuata* | MML0802 | MML | tank | 6/16/2008 | 6/16/2008 | Male | Adult | n |
| *Feresa attenuata* | MML0803 | MML | tank | 6/16/2008 | 6/16/2008 | Male | Adult | n |
| ***Globicephala macrorhynchus*** | **Captain Sully** | **Curacao** | **ocean** | **7/14/2009** | **8/17/2009** | **Male** | **Subadult** | **Y** |
| *Grampus griesus* | Rocky | Riverhead | air | 7/31/2004 | 4/11/2005 | Male | Subadult | n |
| *Grampus griesus* | Bonnie | MML | tank | 7/16/2005 | 8/29/2005 | Female | Adult | n |
| *Grampus griesus* | Clyde | MML | tank | 7/16/2005 | 8/29/2005 | Male | Adult | n |
| *Grampus griseus* | Bam Bam | MML | tank | 5/4/2007 | 6/22/2007 | Male | Calf | n |
| *Grampus griseus* | Betty | MML | tank | 5/4/2007 | 5/5/2007 | Female | Adult | n |
| *Grampus griseus* | Pebbles | MML | tank | 5/4/2007 | 5/5/2007 | Female | Calf | n |
| *Grampus griseus* | Wilma | MML | tank | 5/4/2007 | 5/5/2007 | Female | Adult | n |
| *Mesoplodon europaeus* | Jules | HBOI | tank | 7/20/2004 | 7/22/2004 | Male | Subadult | n |
| *Stenella frontalis* | MMC-Sf-0209 | MMC | Florida Bay | 2/14/2009 | 3/9/2009 | Male | Subadult | n |
| *Stenella longirostris* | Harley | MML | tank | 4/20/2004 | 10/18/2006 | Female | Subadult | n |
| ***Steno bredanensis*** | **JB** | **Gulf World** | **tank** | **2/24/1996** | **3/6/2004** | **Male** | **Adult** | **Y** |
| ***Steno bredanensis*** | **Vixen** | **MML** | **air** | **12/25/2002** | **5/10/2004** | **Female** | **Subadult** | **Y** |
| *Steno bredanensis* | Bashful | MML | air | 8/6/2004 | 10/11/2004 | Female | Adult | n |
| *Steno bredanensis* | Doc | MML | air | 8/6/2004 | 10/11/2004 | Male | Subadult | n |
| *Steno bredanensis* | Dopey | MML | air | 8/6/2004 | 9/21/2004 | Male | Adult | n |
| *Steno bredanensis* | Sleepy | MML | air | 8/6/2004 | 9/21/2004 | Male | Adult | n |
| *Steno bredanensis* | Sneezy | MML | air | 8/6/2004 | 9/21/2004 | Male | Adult | n |
| ***Steno bredanensis*** | **CC224** | **Gulf World** | **tank** | **8/26/2004** | **8/26/2005** | **Male** | **Adult** | **Y** |
| *Steno bredanensis* | GW04008A | Gulf World | tank | 9/27/2004 | 8/26/2005 | Female | Adult | n |
| *Steno bredanensis* | R137 | MMC | Florida Bay | 3/2/2005 | 4/21/2005 | Female | Adult | n |
| *Steno bredanensis* | R372 | MMC | Florida Bay | 3/2/2005 | 4/21/2005 | Female | Adult | n |
| *Steno bredanensis* | R375 | MMC | Florida Bay | 3/2/2005 | 4/21/2005 | Female | Calf | n |
| *Steno bredanensis* | R375 | Gulf World | tank | 3/2/2005 | 8/26/2005 | Female | Calf | n |
| ***Steno bredanensis*** | **Astro** | **Gulf World** | **tank** | **4/18/2005** | **8/26/2005** | **Male** | **Subadult** | **Y** |
| ***Steno bredanensis*** | **Dancer** | **Mote** | **air** | **3/24/2007** | **3/30/2007** | **Female** | **Subadult** | **Y** |
| ***Tursiops truncatus*** | **Panama** | **CMA** | **tank** | **10/21/2000** | **2/27/2009** | **Female** | **Adult** | **Y** |
| ***Tursiops truncatus*** | **Caesar II** | **Mote** | **air** | **3/23/2004** | **5/10/2004** | **Male** | **Adult** | **Y** |
| *Tursiops truncatus* | Indy | CMA | tank | 5/3/2004 | 2/27/2009 | Male | Subadult | n |
| *Tursiops truncatus* | Winter | CMA | tank | 12/10/2005 | 2/26/2009 | Female | Calf | n |
| ***Tursiops truncatus*** | **MML FB303** | **Dolphins Plus** | **air** | **11/12/2006** | **3/9/2009** | **Female** | **Adult** | **Y** |
| ***Tursiops truncatus*** | **MML FB303** | **MMC** | **Florida Bay** | **11/12/2006** | **2/18/2007** | **Female** | **Adult** | **Y** |
| ***Tursiops truncatus*** | **MML0701** | **MML** | **air** | **1/30/2007** | **3/12/2007** | **Female** | **Subadult** | **Y** |
| *Tursiops truncatus* | MML0807 | Mote | tank | 12/16/2008 | 12/23/2008 | Female | Subadult | n |
